# Clinical Thermography of the Diabetic Foot Using a Low-Cost Thermal Camera: Processing and Instrumental Framework

**DOI:** 10.3390/s26082438

**Published:** 2026-04-16

**Authors:** Vanéva Chingan-Martino, Mériem Allali, Stéphane Henri, El Hadji Mama Guène, Dominique Gibert, Antoine Chéret

**Affiliations:** 1 Diabetic Foot Service, University Hospital, 97139 Les Abymes, Guadeloupe, France; 2 INSERM-CIC-1424, University Hospital, 97139 Les Abymes, Guadeloupe, France; antoine.cheret@chu-guadeloupe.fr; 3 Emergency Medical Service (SAMU-SAU), University Hospital, 97139 Les Abymes, Guadeloupe, France; maira.a@hotmail.fr; 4 Artificial Intelligence Lo Health Research & Medical Innovation, 97190 Le Gosier, Guadeloupe, France; stephanehenri7@hotmail.com; 5 Solaymath, Tamusana Senegal, France; elhadjmama.guene@gmail.com; 6 LGL-TPE, Univ Lyon, Univ Lyon 1, ENSL, CNRS, UMR 5276, 69622 Villeurbanne, France; gibert.dominique@gmail.com; 7 Service de Plateforme de Diagnostic et Thérapeutique Pluridisciplinaires, University Hospital, 97139 Les Abymes, Guadeloupe, France

**Keywords:** thermography, diabetic foot, LWIR, radiometry, calibration, NETD, geometric correction, multimodal registration, segmentation

## Abstract

Infrared thermography is a non-contact tool for monitoring inflammatory processes in the diabetic foot, but quantitative bedside use remains challenging with low-cost thermal infrared cameras due to radiometric drift, non-uniformity (vignetting), geometric distortions, and visible–thermal parallax. This paper presents an end-to-end clinical and instrumental framework built around a cheap thermal camera to ensure reproducible acquisition and physically consistent temperature estimation. The approach combines a standardized mobile acquisition setup and measurement protocol, extraction of embedded radiometric data from raw images, radiometric inversion with atmospheric correction, vignette correction performed in the radiometric domain, and geometric calibration of both visible and infrared sensors using dedicated (thermal) calibration targets. Accurate visible–infrared registration is obtained from hybrid heated markers, enabling reliable overlay and downstream analysis. The full processing chain yields quantitative thermograms with radiometric errors below 0.15 °C and sub-pixel multimodal alignment, supporting the detection of clinically relevant plantar temperature asymmetries and paving the way for routine calibrated low-cost thermography in diabetic foot care.

## 1. Introduction

Diabetes mellitus (DM) is a major healthcare issue whose incidence grows rapidly each year. International organizations (e.g., the International Diabetes Federation (IDF)) indicate a global population of DM of 590 million in 2025 with a prediction of 850 million in 2050 [1]. DM provokes various avenues of deterioration in the body like blindness, cardiac illness, chronic kidney disease, neuropathy, and lower-limb ulcers [2]. The most common impact is on the lower limbs and, particularly, the feet, with a high risk of ulcers and lower limb amputations of about 700 amputations out of 100,000 patients each year. An amputation has a high monetary cost and results in a strong reduction in the quality of life. International healthcare organizations concentrate on the prevention of diabetic foot [3] and advise feet evaluations with healthcare professionals at least once a year, and encourage patients to perform daily inspections of their feet so they can identify risk factors and reduce the rate of ulcer complications by expediting professional treatment. An early detection of any abnormality is essential to avoid an amputation.

Among prevention and assessment methods, infrared thermography is attracting increasing interest for the early detection of inflammatory processes in the diabetic foot [4,5,6]. Recent bibliometrics [7] identifies more than 300 relevant publications and confirms a doubling of publications on diabetic foot thermography between 2018 and 2025 (from 20 to >40 articles/year), reflecting a renewed clinical interest marked by the rise of AI [8,9,10,11]. This growing body of work underscores the clinical relevance of thermography for early detection and prevention of diabetic foot complications through temperature monitoring strategies. In particular, a bilateral temperature difference greater than approximately 2 °C suggests a likely infection [12]. Highly cited studies emphasize the role of regular skin temperature assessment in reducing ulceration risk, reflecting a strong shift toward preventive approaches in diabetic foot management. Furthermore, the literature reveals a predominance of clinical studies and significant international collaboration, highlighting both the translational focus and the global interest in this technology.

The renewed interest in thermography is primarily due to significant advancements in thermal imaging cameras operating in the 8 μm to 14 μm Long-Wavelength Infra-Red (LWIR) range [13]. These cameras utilize uncooled microbolometers, are becoming increasingly affordable, and offer a resolution of approximately 50 mK to 100 mK. Easy to use, these cameras are increasingly being employed in exploratory clinical trials and will eventually become commonplace in the medical field [14]. The use of thermography has recently been further stimulated by the introduction of low-cost cameras with the accuracy required to identify medically relevant areas of thermal anomaly. Examples of such cameras include the FLIR (27700 SW Parkway Ave. Wilsonville, OR 97070, USA) Lepton [15] and the FLIR C5 [16], the latter being used in the present study.

An important point to consider for the widespread use of thermography in diabetic foot diagnosis is the need to integrate this method into a multimodal approach. Recent studies [17,18] have demonstrated the value of combining LWIR images with visible (VIS) images, enabling dermoscopic analysis for the precise detection of lesions, calluses, and fissures [19,20]. This topic is addressed in the present study, where we propose both a hardware solution and an algorithmic solution to obtain an accurate overlay of LWIR and VIS images. This allows the physician to simultaneously interpret both types of images thanks to a geometric correspondence between the thermal structures and the structures in the visible image.

However, despite these technological advances, the clinical use of thermography remains limited by important variability in measurements and interpretation. In practice, thermal images can be significantly influenced by several factors such as ambient temperature, airflow, patient positioning, acclimatization time, skin conditions (e.g., moisture or callus), and camera-to-subject distance. These factors can lead to substantial variations in recorded temperatures, sometimes comparable in magnitude to clinically relevant thresholds such as the 2 °C bilateral difference. As a result, the lack of standardized acquisition protocols can compromise the reliability and reproducibility of measurements, making bedside interpretation difficult and potentially leading to inconsistent clinical decisions. These limitations are not restricted to low-cost devices but are inherent to thermographic assessment itself, even when using advanced systems. While higher-end cameras improve thermal sensitivity and spatial resolution, they do not eliminate the influence of environmental and procedural factors. Consequently, without rigorous control of acquisition conditions, variability remains a major obstacle to routine clinical adoption [21].

To be useful and operational in clinical applications, procedures must take into account several constraints imposed by hospital conditions and the organization of diabetic foot care units. In particular, thermographic measurements must be integrated into a care pathway and, as such, must be rapid and performed in a minimally controlled environment very different from laboratory conditions. All measurement procedures must be simple while guaranteeing the required quality. These procedures constitute a coherent and sequential set whose objective is to produce a file attached to each patient containing all the information necessary for subsequent interpretative analyses. The objective of the present paper is to detail such a set of procedures developed jointly by the medical team and physicists and implemented in the Diabetic Foot Service of our University Hospital on a large scale with cohorts of several dozen of diabetic patients.The clinical results concerning these cohorts will be detailed in a separate article.

The article is organized as follows:

- Section 2 gives a description of a setup based on the low-cost FLIR C5 thermal camera and designed to perform standardized thermography acquisition at the patient’s bedside.
- In the Measurement Protocol Section, we enumerate the stages of our elaborated protocol to examine cohorts of dozens of patients in a real hospital setting.
- Section 3 presents the method used to extract the metadata and the radiometric images from the jpeg thermographic images produced by the camera. This stage is mandatory to obtain the raw data necessary to apply the processing stages described in the next sections.
- In Section 4 we address the very important issue of vignetting correction of the radiometric data. This correction is essential to derive corrected temperature images, allowing a reliable clinical interpretation of temperature differences between the two feet.
- Section 5 presents the first processing step towards the fusion of both the visible and temperature images. This fusion is our second main processing chain whose objective is to obtain pairs of accurately superimposed visible/temperature images such that physicians can establish reliable correspondence between anatomical features visible in both type of images. We describe an easy-to-construct thermal checkerboard device that corrects geometric distortions in both types of images before they are superimposed.
- In Section 6 we give a detailed description of the processing stages for the fusion of the visible/temperature images. We present a hardware device with thermal markers visible in both types of images, used to establish a geometric registration model of the visible image onto the temperature image. This results in a very good superimposition of both images that can therefore be reliably analysed by the physicians.
- The article concludes with a discussion and a conclusion. The algorithms developed in this work are described in the Appendices Appendix A, Appendix B, Appendix C and Appendix D.

## 2. Acquisition Setup

**Figure 1:**
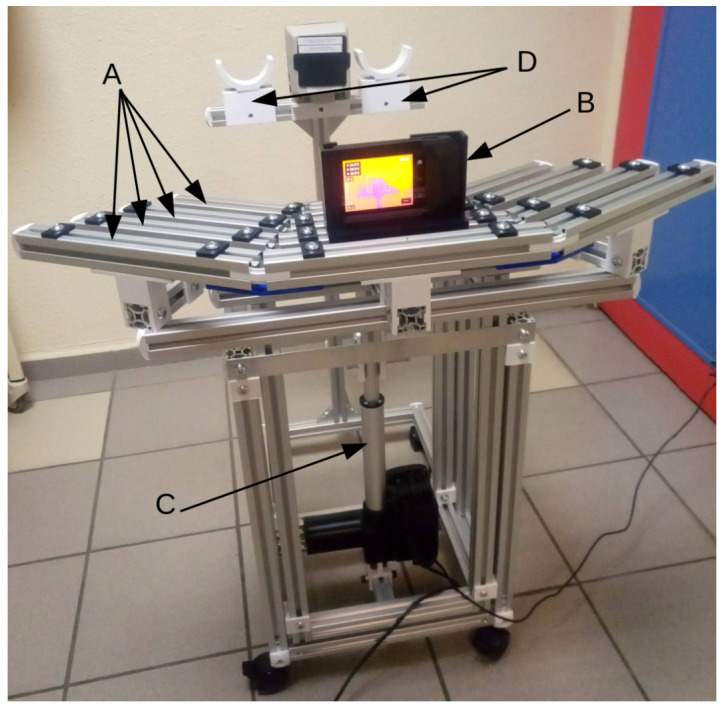
Overview of the acquisition frame with the FLIR C5 thermal imaging camera and its case. The device features stepped guide rails arranged like an amphitheatre (A), on which the camera guide (B) can be precisely positioned to capture images at known angles and distances. An electric actuator (C) adjusts the height of the guide rails to the patient’s bed. Footrests (D) allow for comfortable and stable positioning of the patient’s feet. The entire unit is equipped with casters for easy transport to the patient’s bedside, and its compact size allows it to pass through standard hospital doorways. An optional battery power bank provides independent electrical operation for the actuator. The device is positioned at the foot of the patient’s bed so that their ankles can be placed on the footrests. The camera and its case are placed on the guide rails at the optimal distance so that the patient’s feet occupy most of the field of view of the LWIR camera.

The acquisition device was designed to be compact, mobile, and compatible with bedside use in routine clinical settings (Figure 1). The entire acquisition setup was designed and fabricated by us. The system can be easily transported between patient rooms and is dimensioned to pass through standard hospital doorways. A dedicated mobile frame was developed, incorporating guide rails that allow precise and reproducible positioning of the thermal camera in terms of angle and distance relative to the patient’s feet. Curved footrests of various sizes are positioned in front of the camera to ensure consistent and comfortable foot placement. The guide rails can be adjusted vertically using a motorized actuator to center the plantar surfaces within the field of view. A reference blackbody is placed between the footrests for radiometric calibration. The entire system is powered by an integrated power bank, ensuring full electrical autonomy and facilitating deployment at the bedside without additional infrastructure.

**Figure 2:**
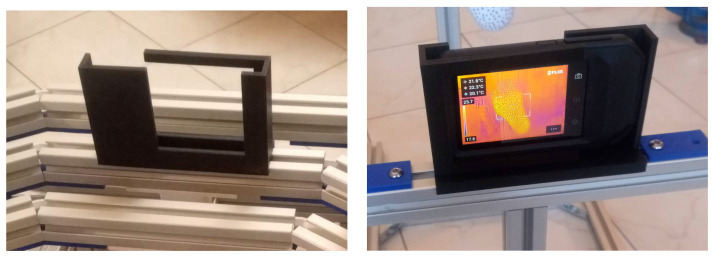
(**Left**): FLIR C5 thermal camera guiding sleeve used to firmly secure the camera in a reproducible manner (**Right**): FLIR C5 camera inserted in the guiding sleeve and ready for image acquisition. The two blue plates fixed to the guiding rail act as stops to limit the camera’s translational movement.

The thermal camera used in this study is the FLIR C5 model [22], selected for its portability, ease of use, and low cost, making it suitable for routine clinical practice, including in resource-limited settings. This type of equipment can be used in small hospital settings and in the offices of private doctors and nurses. The device integrates a 5-megapixel visible (VIS) camera (field of view 71.5°×56°) and a 160×120 pixel uncooled microbolometer (NETD < 70 mK; field of view 54°×42°), operating in the 8 μm to 14 μm (LWIR) spectral range [22]. A dedicated guide sleeve (Figure 2 left) ensures fixed and reproducible positioning of the camera on the guide rails (Figure 2 right). In their recent study on a cohort of patients, Marcon Alfieri et al. [16] show that the FLIR C5 camera is suitable to detect temperature differences of about ≃0.2 °C. Although higher-end systems exist, this configuration was chosen to reflect realistic clinical deployment conditions. Importantly, the study design accounts for known sources of variability in thermographic measurements, regardless of device cost, by enforcing standardized acquisition conditions.

**Figure 3:**
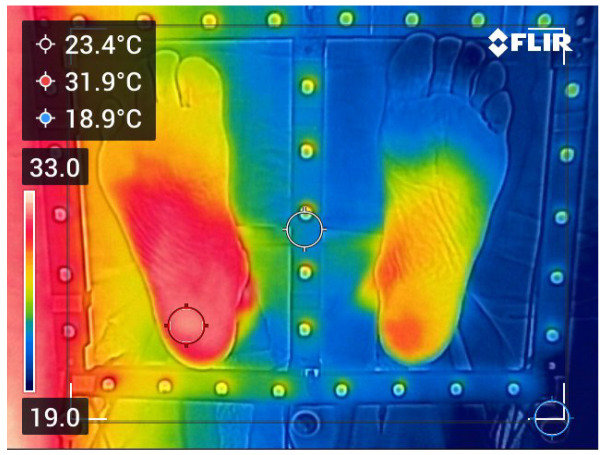
Example of raw thermography obtained in real time during a healthy volunteer examination. Note the huge horizontal temperature gradient caused by vignetting artefact and discussed in Section 4. Such a gradient must absolutely be corrected so that small temperature differences between each foot can be medically interpreted. Data acquisition conditions identical to those implemented in a clinical setting: duration of acclimatization 30 min, legs horizontal and feet dangling in the air, room temperature 22.4 °C and relative humidity 58%, distance between camera and feet 58 cm.

The FLIR C5 camera simultaneously captures a VIS and a LWIR image that can be merged to give a FLIR MSX^®^ [23] image where sharp details of the VIS image are superimposed on the LWIR thermography to enhance anatomical regions (Figure 3). Images such as the one shown in Figure 3 constitute the raw thermographies collected during bedside acquisition.

### Measurement Protocol

To ensure reproducibility while maintaining compatibility with routine care, a standardized bedside workflow was implemented. The procedure is designed to be performed within a typical diabetic foot assessment, without disrupting clinical organization, and can be conducted by trained nursing staff with minimal additional workload. The workflow is as follows:

■ Preparation before receiving patients
  1. The camera and the reference blackbody are powered-on approximately half an hour before the start of measurements.
  2. When available, the thermal markers used for post-processing image registration (see Section 6) are powered-on to warm up.
  3. The camera setting is controlled, particularly the date time and the measurement distance, $d_{o}$, which must be set to 50 cm. These data will later be retrieved in the metadata and used for further processing. The emissivity is set to ϵ=0.98 for human skin in the LWIR range [24,25].
■ Actions in the presence of the patient
  1. The patient is placed at rest, lying on their back and barefoot, in the room where the measurements are taken to reach thermal equilibrium. This rest period has a duration of at least 15 min, but preferably 30 min [26].
  2. The patient identification label (e.g., a barcode) is placed between the footrests, within the field of view of the VIS camera. This step is extremely important to ensure data traceability and avoid attribution errors.
  3. The nurse records the air temperature and the relative humidity in the room. These data are reported on the patient’s identification label together with the date time.
  4. The nurse ensures that the patient’s feet are dry and mentions the presence of wet wounds if there are any.
  5. The rest period is used by the nurse to check the patient identification label and conduct the patient’s medical interview and examination.
  6. The physician performs several thermographic scans at different distances and angles, depending on the patient’s needs.

This structured workflow ensures that thermographic acquisition is rapid (a few minutes per patient), reproducible, and feasible in real-world bedside conditions, without requiring highly controlled environments. The use of the positioning frame and standardized protocol directly addresses known sources of variability (environmental, technical, and operator-dependent), thereby improving the consistency of measurements. We draw the reader’s attention to the importance of the identification label, which must be carefully checked to avoid any attribution error. At the end of the patient measurement session, we have a set of thermographic images like the one in Figure 3. These thermographies are the default ones provided by FLIR and require several processing steps before they can be interpreted by physicians.

## 3. Raw Data Processing

The raw images obtained from thermography sessions with patients are far from being directly usable by physicians for interpretation. The raw image shown in Figure 3 exhibits several vignetting defects that must be corrected to obtain a quantitatively interpretable thermography. For example, a significant horizontal gradient is observed due to a misalignment of the microbolometer array relative to the camera’s optical axis. The correction of vignette artefacts is detailed in Section 4. The overlay of the VIS image onto the thermography is imperfect due to parallax effects caused by the short distance between the camera and the patient’s feet. This defect is compounded by the fact that the geometric distortions of the VIS and LWIR cameras differ. The corrections of geometric distortions and VIS and LWIR image registration are described in Section 5.

### Decoding Raw Images and Patient’s Record Initialization

To perform the corrections listed above, access to the basic data is necessary. Fortunately, these data are actually encoded in the raw JPEG images and can be retrieved using tools like EXIF Tools. Numerous parameters can be extracted from the raw images, including the VIS and LWIR radiometric images, as well as the parameters for converting radiometric data to temperature. Other parameters (serial number, camera model, distance setting, timestamp, etc.) are also extracted and saved in the patient’s file.

A set of MATLAB v2024a code was written to perform these tasks and sequentially build the patient’s record. This record is contained in a .mat file, which includes several structures that are progressively enriched as the data is processed and analysed. Each structure contains essential data such as the patient’s name, the date of the examination, and the names of the physicians and other personnel involved in the process. Our experience with several hundred patients shows that this approach is efficient and reliable. The use of structures is well-suited to the evolving nature of patient records (new examinations, repeat analyses, etc.).

The MATLAB .mat file does not comply with the DICOM standard format often used in hospital information systems [27]. The reason for our choice is that although the DICOM standard includes a specific “TG” (0008,0060) modality for thermography, it is not yet accompanied by recommendations regarding the Information Object Definitions (IODs) to be used for thermographic data. Until the “TG” modality is fully defined and validated by the DICOM committees, database management and visualization software will not incorporate this thermography-specific modality. It is hoped that this situation will change in the coming years [28].

## 4. Correction of Vignetting Artefacts in the Radiometric Data

LWIR images can be affected by significant vignetting [29] which, in the example of Figure 3, appears as a strong gradient superimposed on the real thermal field. In this example, this long-wavelength non-uniformity is likely to be caused by a lack of parallelism between the focal plane and the camera’s array of microbolometers.

Vignetting is primarily caused by optical effects (lens transmission, angular response) and detector non-uniformities, which act on the radiometric signal before any physical inversion is applied. Consequently, vignetting is intrinsically a multiplicative effect in the radiometric domain. Correcting it directly on the radiometric data preserves the physical structure of the acquisition chain and avoids mixing spatial non-uniformities with the non-linear temperature inversion.

In contrast, temperature images are obtained after applying a non-linear transformation (Planck inversion combined with internal camera corrections). In this domain, a purely multiplicative radiometric bias no longer remains strictly multiplicative and may become temperature-dependent or partially additive. Performing vignetting correction after temperature conversion can thus lead to level-dependent residuals and over- or under-correction, especially when scenes span several degrees.

For these reasons, vignetting correction is best performed on radiometric data, prior to temperature computation. The corrected radiometric images can then be converted into temperature in a consistent and physically meaningful manner.

Vignetting varies from one camera to another and may slightly change over time for a given camera. Correcting them is essential for obtaining interpretable thermographies and this can only be achieved through an experimental procedure in which a camera-specific model is constructed as described below. More generally, non-uniformity correction methods continue to evolve, including locally adaptive approaches developed for uncooled IR cameras [30]. More generally, non-uniformity correction methods continue to evolve, including locally adaptive approaches developed for uncooled IR cameras [30].

### 4.1. Construction of Smooth Flat-Fields (Algorithm A1)

In the radiometric space, the vignetting effects are modelled as a slowly varying gain field G(x,y,t)>0 such that,

(1) $$R\left(x,y,t\right)\;=\;G\left(x,y,t\right)\;R^{★}\left(x,y,t\right),$$

where R(x,y,t) is the measured radiometric signal at pixel (x,y) and time *t*, and $R^{★}$ is the vignette-free radiometric image. The objective is to compute a model $\hat{G}\left(x,y,t\right)$ of the gain from flat-field sequences acquired at multiple epochs to obtain corrected radiometric images:

(2) $$\hat{R^{★}}\left(x,y,t\right)\;=\;\frac{R(x,y,t)}{\hat{G}\left(x,y,t\right)}.$$

For a given camera, flat-field thermograms are acquired at *K* distinct epochs, ${\left\{t_{k}\right\}}_{k=1}^{K}$, under constant geometry, fixed focus, and stable scene conditions. In practice, flat-field measurements may simply be done by putting the camera against cardboard. At each epoch $t_{k}$, a sequence $F_{k}$ of $N_{k}$ flat-field radiometric frames is recorded:

(3) $$F_{k}\;=\;{\left\{F_{n}\left(x,y,t_{k}\right)\right\}}_{n=1}^{N_{k}}.$$

The flat-field scene is intended to be spatially uniform, so that the spatial content of each $F_{n}$ contains only vignetting and noise.

To reduce measurement noise and mitigate sporadic outliers (hot pixels, transient reflections), we first compute a robust flat image for each sequence,

(4) $$\bar{F}\left(x,y,t_{k}\right)\;=\;\mathrm{median}\left[F_{1}\left(x,y,t_{k}\right),F_{2}\left(x,y,t_{k}\right),\ldots ,F_{N_{k}}\left(x,y,t_{k}\right)\right],$$

where the pixelwise median is preferred to the mean for robustness.

Optionally, extreme values can be clipped using low/high quantiles of ${\bar{F}}_{k}$ to further reduce the influence of rare artefacts. In practice we used a symmetric quantile clamp, e.g., $\left[q_{\mathrm{lo}},q_{\mathrm{hi}}\right]=\left[0.02,0.98\right]$,

(5) $$\bar{F}\left(x,y,t_{k}\right)\leftarrow \min\;\left[\max\left(\bar{F}\left(x,y,t_{k}\right),Q_{q_{\mathrm{lo}}}\right),Q_{q_{\mathrm{hi}}}\right],$$

with $Q_{q}$ the *q*-quantile of ${\bar{F}}_{k}\left(x\right)$ over all pixels.

Some more noise reduction is obtained by applying a Gaussian smoothing to $\bar{F}\left(x,y,t_{k}\right)$,

(6) $$\tilde{F}\left(x,y,t_{k}\right)\;=\;\left(\bar{F}\left(\;\cdot \;,\;\cdot \;,t_{k}\right)*G_{\sigma }\left(\;\cdot \;,\;\cdot \;\right)\right)\left(x,y\right),$$

where $G_{\sigma }$ is a 2D Gaussian kernel with standard deviation σ (in pixels) and ∗ stands for 2D convolution. In order to preserve the spatial structure of the gain, σ is in the range 3–6 pixels. The smoothed field $\tilde{F}$ captures both the multiplicative shape and an arbitrary global scale from which the multiplicative gain is derived.

### 4.2. Derivation of Multiplicative Gain Fields

To define a gain field with unit global level, we normalize the smoothed field by a robust scalar reference,

(7) $$\hat{G}\left(x,y,t_{k}\right)\;=\;\frac{\tilde{F}\left(x,y,t_{k}\right)}{\langle \tilde{F}\left(x,y,t_{k}\right)\rangle },$$

where $\langle \tilde{F}\rangle$ stands for either the mean or the median. This yields $\langle {\hat{G}}_{k}\rangle \approx 1$ and ensures that applying Equation (2) does not alter the global radiometric scale on average.

**Figure 4:**
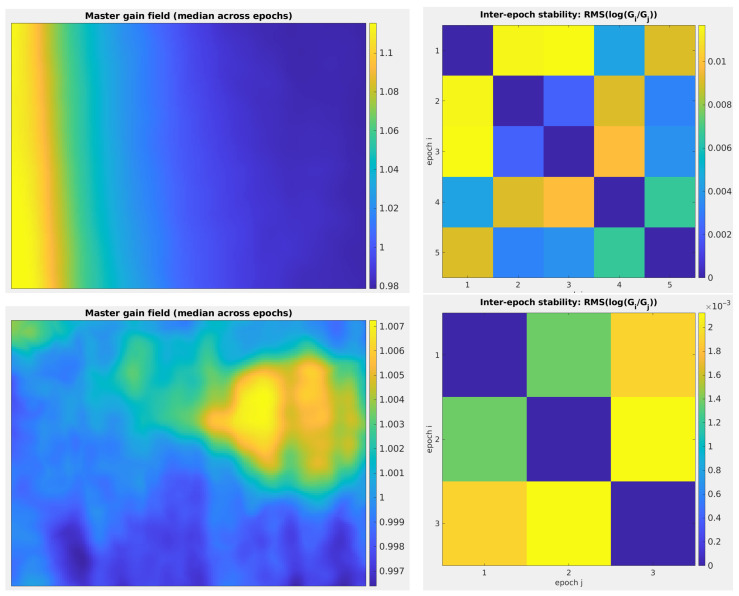
(**Left**): Examples of a master gain field ${\hat{G}}_{\mathrm{master}}$ given by Equation (8) for two different FLIR C5 cameras. (**Right**): Corresponding inter-epoch root-mean-square ${\mathrm{RMS}}_{i,j}$ given by Equation (10). Observe the huge difference in the master gain amplitude between the two cameras. This illustrates the wide variability in quality among low-cost cameras.

When the vignetting pattern is stable across epochs, a single master gain field can be computed as the pixelwise median across epochs (left of Figure 4):

(8) $${\hat{G}}_{\mathrm{master}}\left(x,y\right)\;=\;\mathrm{median}\left[\hat{G}\left(x,y,t_{1}\right),\hat{G}\left(x,y,t_{2}\right),\ldots ,\hat{G}\left(x,y,t_{K}\right)\right].$$

To quantify the stability of the gains obtained for all epochs, we compute inter-epoch discrepancies in the log-gain domain, which is a natural measure for multiplicative effects. For epochs $t_{i}$ and $t_{j}$,

(9) $$D_{i,j}\left(x,y\right)\;=\;\log\hat{G}\left(x,y,t_{i}\right)-\log\hat{G}\left(x,y,t_{j}\right)\;=\;\log\;\left(\frac{\hat{G}\left(x,y,t_{i}\right)}{\hat{G}\left(x,y,t_{j}\right)}\right),$$

and we report a scalar summary such as the root-mean-square (RMS) (right of Figure 4):

(10) $${\mathrm{RMS}}_{i,j}\;=\;\sqrt{\frac{1}{N_{x}\times N_{y}}\sum_{x,y}D_{i,j}{\left(x,y\right)}^{2}}.$$

Small values of ${\mathrm{RMS}}_{i,j}$ and high correlation between $\log{\hat{G}}_{i}$ and $\log{\hat{G}}_{j}$ indicate stable vignetting, supporting the use of ${\hat{G}}_{\mathrm{master}}$. The inter-epoch discrepancies of Figure 4 (right) are very small, indicating that the master gain may safely be used. To avoid numerical instabilities, a small positive floor ε≪1 (e.g., $10^{-6}$ in normalized units) is applied to $\hat{G}$,

(11) $$\hat{G}\left(x,y,t_{k}\right)\leftarrow \max\left(\hat{G}\left(x,y,t_{k}\right),\varepsilon \right).$$

The corrected radiometric image is obtained via elementwise division,

(12) $$\hat{R^{★}}\left(x,y,t_{k}\right)\;=\;\frac{R(x,y,t_{k})}{\hat{G}\left(x,y,t_{k}\right)}.$$

**Figure 5:**
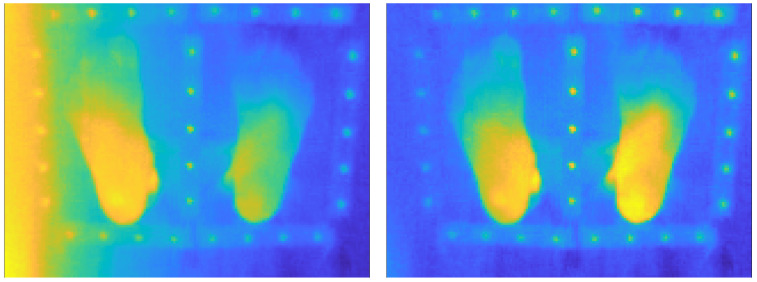
(**Left**): Example of a vignetted radiometric image *R*. (**Right**): Corrected radiometric image $\hat{R^{★}}$ obtained through Equation (12) using the master gain $\hat{G}$ of Figure 4 (top left).

Figure 5 illustrates an example of vignetting correction in the case of a particularly pronounced alteration due to a misalignment of the optical-detector assembly (see Figure 4, top left). Even in this difficult case (in practice, a camera with such a defect should not be used in clinical applications), the vignetting correction procedure proposed in this study successfully restores an image with a flat background.

### 4.3. Temporal Interpolation in the Log-Gain Domain (Algorithm A2)

In the rare cases where the inter-epoch RMS matrix (Figure 4) indicates a non-stationarity which prevents the use of the master gain ${\hat{G}}_{\mathrm{master}}\left(x,y\right)$, it may be useful to use the gains at different epochs, $\hat{G}\left(x,y,t_{k}\right)$, to interpolate a gain model at the time $t_{m}$ of the measurements to be corrected. Because vignetting is multiplicative, interpolation is performed in the log domain to preserve positivity and multiplicative structure. Let *t* fall between two consecutive calibration epochs $t_{j}\leq t_{m}\leq t_{j+1}$. We define the normalized interpolation weight

(13) $$w\;=\;\frac{t_{m}-t_{j}}{t_{j+1}-t_{j}}\in \left[0,1\right],$$

interpolate the log-gain fields linearly:

(14) $$\log\hat{G}\left(x,y,t\right)\;=\;\left(1-w\right)\;\log\hat{G}\left(x,y,t_{j}\right)\;+\;w\;\log\hat{G}\left(x,y,t_{j+1}\right),$$

and exponentiate to obtain the gain:

(15) $$\hat{G}\left(x,y,t\right)\;=\;\exp\;\left(\left(1-w\right)\;\log\hat{G}\left(x,y,t_{j}\right)+w\;\log\hat{G}\left(x,y,t_{j+1}\right)\right).$$

This is equivalent to a geometric interpolation:

(16) $$\hat{G}\left(x,y,t\right)\;=\;\hat{G}{\left(x,y,t_{j}\right)}^{\;1-w}\;\hat{G}{\left(x,y,t_{j+1}\right)}^{\;w},$$

which is the natural interpolation rule for multiplicative fields.

### 4.4. Radiometric-to-Temperature Conversion

The conversion of the corrected radiometric image, $\hat{R^{★}}$, to temperature, *T*, in Celsius units, is performed according to Minkina and Dudzik [31]:

(17) $$T\;=\;P_{B}\times {\left[\log\left(\frac{P_{R1}}{P_{R2}\left(R_{\mathrm{object}}+P_{O}\right)}+P_{F}\right)\right]}^{-1}-273.15,$$

where $P_{B}$, $P_{R1}$, $P_{R2}$, $P_{O}$ and $P_{F}$ are parameters extracted from the FLIR raw image, and the object radiance

(18) $$R_{\mathrm{object}}\;=\;\frac{\hat{R^{★}}}{\epsilon \times \tau \times W_{t}}-R_{\mathrm{atm}}-R_{\mathrm{opt}}-R_{\mathrm{refl}},$$

with ϵ being the emissivity of the object and $W_{t}$ the camera window transmission coefficient, both of which are extracted from the FLIR raw image. The atmosphere transmission (Minkina and Dudzik ([31], p. 163))

(19) $$\tau \;=\;X_{\mathrm{atm}}\times \exp\left[-\sqrt{d_{o}}\left(\alpha_{1}+\beta_{1}\sqrt{W_{p}}\right)\right]+\left(1-X_{\mathrm{atm}}\right)\times \exp\left[-\sqrt{d_{o}}\left(\alpha_{2}+\beta_{2}\sqrt{W_{p}}\right)\right],$$

where $X_{\mathrm{atm}}$, $\alpha_{1}$, $\alpha_{2}$, $\beta_{1}$ and $\beta_{2}$ are constants extracted from the FLIR raw image and the water vapour pressure (Minkina and Dudzik ([31], p. 163)):

(20) $$W_{p}\;=\;RH\times \exp\left(1.5587+0.06939T_{a}-0.00027816T_{a}^{2}+0.00000068455T_{a}^{3}\right).$$

Note that in this last equation, the atmosphere temperature must be given in Celsius units.

The terms $R_{\mathrm{atm}}$, $R_{\mathrm{opt}}$ and $R_{\mathrm{refl}}$ in Equation (18) respectively correspond to the radiance of the atmosphere, the radiance of the transmission optics and the reflected radiance coming from the object. Their expressions are given by:

(21) $$R_{\mathrm{atm}}\;=\;\frac{1-\tau }{\epsilon \times \tau }\times \left[\frac{P_{R1}}{P_{R2}\left(\exp\left(P_{B}/T_{a}\right)-P_{F}\right)}-P_{O}\right],$$

(22) $$R_{\mathrm{opt}}\;=\;\frac{1-W_{t}}{\epsilon \times \tau \times W_{t}}\times \left[\frac{P_{R1}}{P_{R2}\left(\exp\left(P_{B}/T_{w}\right)-P_{F}\right)}-P_{O}\right],$$

and

(23) $$R_{\mathrm{refl}}\;=\;\frac{1-\epsilon }{\epsilon }\times \left[\frac{P_{R1}}{P_{R2}\left(\exp\left(P_{B}/T_{r}\right)-P_{F}\right)}-P_{O}\right],$$

where the temperatures in these last three equations are given in K units.

At the completion of the processing stage, both the corrected radiometric image $\hat{R^{★}}$ and the temperature *T* image are stored in the patient’s record together with meta information describing the processing steps applied to the raw data.

**Figure 6:**
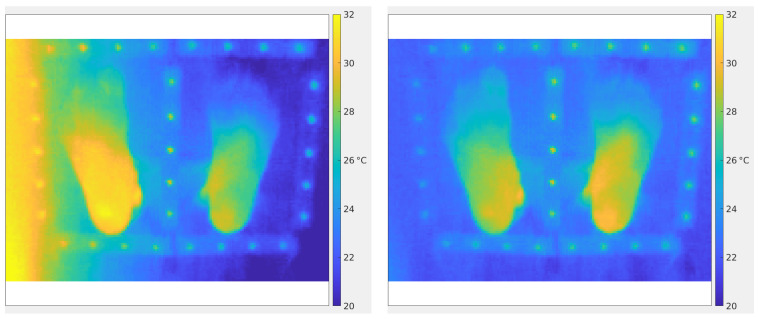
(**Left**): Thermography corresponding to the vignetted radiometric image of Figure 5 (left). (**Right**): Thermography corresponding to the corrected radiometric image of Figure 5 (right). Color scales are the same in both thermographies.

Figure 6 shows the importance of the vignetting correction to obtain as-reliable-as-possible thermographies for medical interpretation. Even if the cameras used for patient examinations never have such a huge vignetting as the one in Figure 5 (left), it is mandatory to always apply the vignetting correction described in this Section. It is also recommended to periodically perform flat-field measurements to assess the stability of the gains $\hat{G}\left(x,y,t_{k}\right)$.

## 5. Correction of Geometric Distortions

The VIS and LWIR images are affected by geometric distortions caused by the optics of the FLIR C5 camera. These distortions must be corrected in order to superimpose the two types of images during the interpretation phase performed by physicians. Geometric corrections are well-known and described in the computer vision literature [32]. The standard procedure involves taking a series of images of a checkerboard pattern from different angles and distances to determine the camera’s intrinsic parameters for correcting the distortions. In our case, we used the MATLAB “Image Processing” toolbox to perform this task.

**Figure 7:**
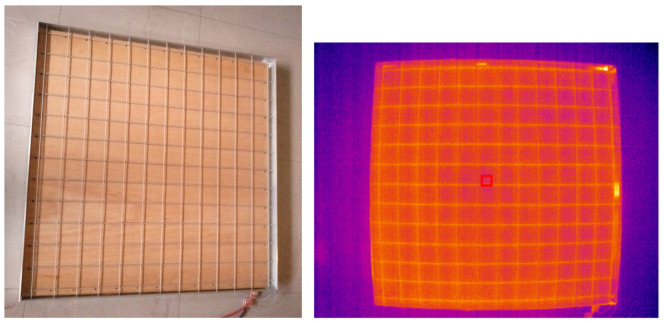
(**Left**): Thermal grid used to calibrate the LWIR camera. (**Right**): LWIR image of the calibration thermal grid. Observe the strong barrel distortion to be corrected.

In the present study, the only unusual aspect of this data processing step concerns the checkerboard pattern used for the LWIR camera. The black and white checkerboard pattern used for the VIS camera does not produce correct images with the LWIR camera. To obtain high-contrast images, we created a thermal checkerboard pattern consisting of a single electrical wire stretched to form a grid (Figure 7, left). By heating the wire with a weak electric current, it becomes clearly visible in LWIR images (Figure 7, right). From our experience, we confirm that this type of technical solution is by far the easiest to implement and the most reliable [33]. The same procedure used for the VIS camera is applied to this checkerboard pattern to determine the intrinsic parameters of the LWIR camera.

The undistorted VIS and LWIR images are written in the patient’s record together with meta information describing the processing steps applied to the initial images.

## 6. Registration of VIS and LWIR Images

Our clinical studies on patient cohorts have highlighted the importance of superimposing the VIS and LWIR images as precisely as possible. This is crucial for facilitating image interpretation by physicians. Physicians must be able to locate a point on one image with a precise corresponding point on the other. Therefore, we have paid particular attention to this issue, both in terms of hardware and data analysis tools as described below.

Figure 3 shows that the FLIR MSX^®^ [23] superposition performed in the FLIR C5 camera is not sufficiently accurate for our purpose. This is due to significant parallax effects caused by the short distance between the camera and the patient’s feet (approximately 50 cm). To obtain a good overlay of the VIS image onto the LWIR image, a sophisticated transformation is necessary because the parallax effects depend quite strongly on the actual distance between the camera and each point in the VIS image. This is evident in Figure 3, where the shift observed on the reference black body in the foreground differs from the shift observed on the patient’s head in the background. The only reliable way to address this problem is to use pairs of homologous points in both images to calculate an experimental transformation function that optimally distorts the VIS image.

### 6.1. Usage of Hybrid Visible and Thermal Markers

At the beginning of our study, we used a procedure involving manually identifying homologous points in both the VIS and LWIR images to determine the transformation parameters to be applied. Since registration must be perfect at the patient’s feet, the homologous points were primarily located around the feet in the foreground of the image. Besides the fact that this procedure is time-consuming, it is sometimes very difficult to find reference points in LWIR images for diabetic patients with very poor blood circulation in certain parts of the foot. In such cases, the absence of homologous points in some parts of the images biases the transformation and leads to poor superposition of the VIS and LWIR images.

To improve and automate the detection of homologous point pairs, we designed thermal markers visible in both VIS and LWIR images (Figure 3). The device used in this study consists of an assembly of black strips containing electrical resistors (10 Ω and 5 W) that heat up sufficiently to be clearly visible in the LWIR image. A 10 mm diameter circular hole is drilled in front of each resistor to expose a portion of the hot element. In this way, each resistor produces a 10 mm white disk surrounded by a black margin in the VIS images and a hot disk in the LWIR images. The strips were covered with matte black adhesive tape to prevent reflections in the VIS images.

### 6.2. Detection of White Marker in VIS Images

Automatic detection with the MATLAB function imfindcircles from the “Image Processing” toolbox is not reliable enough in our case, and we developed a dedicated method that explicitly shows the strong contrast between white disks and their black neighbourhood. The proposed detection method is formulated independently of any global geometric structure in the scene, ensuring robustness to illumination gradients and partial occlusions.

The input color image is first converted to a greyscale image I(x,y) and a slowly varying background component is estimated by applying a large-scale Gaussian filter and subtracted from the original image. This operation compensates for low-frequency illumination variations and enhances the local contrast of small bright structures. For each pixel position (x,y) in the image, two local intensity averages are computed:

- The mean intensity $\mu_{\mathrm{disk}}\left(x,y\right)$ inside a disk $D_{r}\left(x,y\right)$ of radius r≈5 pixels centred at (x,y) and corresponding to the expected size of the target;
- The mean intensity $\mu_{\mathrm{ring}}\left(x,y\right)$ inside an annular region $R_{r,w}\left(x,y\right)$ centred at (x,y), with inner radius *r* and outer radius r+w (w≈5 pixels), and representing the immediate local background.

A local photometric score is then defined as the difference between the disk and ring averages:

(24) $$S\left(x,y\right)=\mu_{\mathrm{disk}}\left(x,y\right)-\mu_{\mathrm{ring}}\left(x,y\right).$$

This score takes high positive values when a bright circular region is surrounded by a significantly darker neighbourhood, which corresponds to the expected appearance of a valid target. Candidate target centres are identified as local maxima of the score map S(x,y). Additional constraints on the absolute brightness of $\mu_{\mathrm{disk}}$ and the darkness of $\mu_{\mathrm{ring}}$ are used to reject ambiguous structures.

Each candidate center is further validated by a local geometric analysis. A region of interest centred on the candidate is segmented using an adaptive threshold to extract the bright region. The connected component containing the candidate center is retained and characterized using standard geometric descriptors such as area, perimeter, and the lengths of the major and minor axes.

A candidate is accepted as a valid target if:

- Its area is consistent with that of a disk of radius *r*;
- Its circularity, defined as $4\pi A/P^{2}$, exceeds a prescribed threshold;
- The ratio between the minor and major axes is close to unity.

For each validated target, the center position is retained and the radius is estimated from the equivalent disk area. This area-based estimation is stable even in the presence of slight shape distortions or pixel-level aliasing. Because the detection relies solely on local photometric contrast and geometric consistency, it does not require explicit detection of global dark supports and remains robust to changes in orientation, partial occlusions, and moderate illumination variability. The use of a uniform matte black background enhances the reliability of the method by stabilizing the ring intensity statistics.

In practice, the markers are positioned in a plane passing through the soles of the patient’s feet. In addition to serving as reference points, the 10 mm diameter circles act as a spatial scale for measuring distances and areas on the patient’s feet.

### 6.3. Robust Detection of Thermal Marker Imprints in LWIR Images (Algorithm A3)

**Figure 8:**
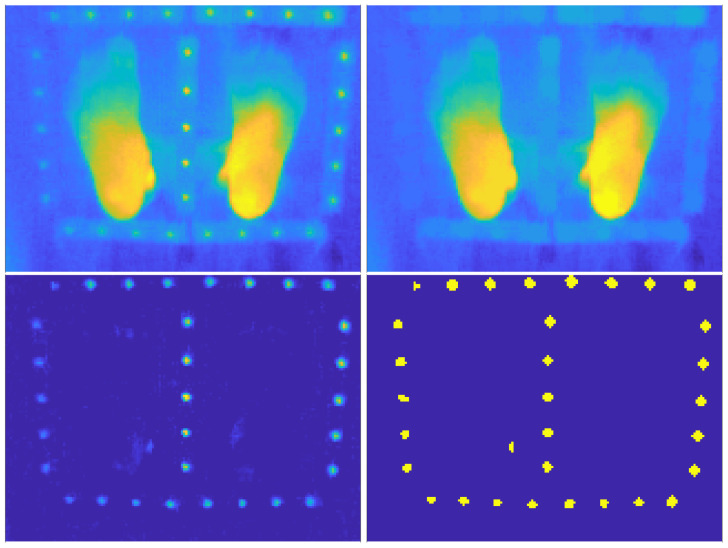
(**Top left**): Initial temperature image *T* corrected for vignetting. (**Top right**): Temperature background image $T_{\mathrm{bk}}$. (**Bottom left**): Temperature relief image $T_{\mathrm{markers}}$ obtained by removing the background image from the initial temperature image. (**Bottom right**): Binary mask corresponding to the retained thermal marker patches $B_{\mathrm{markers}}$.

The detection of the thermal markers in the LWIR image (Figure 6, right) is a little bit tricky because their appearance in the LWIR image significantly departs from that of a uniform disk. The thermal footprint of the markers is best described as a localized thermal hill characterized by a single maximum and diffuse boundaries. The objective is to robustly identify isolated local maxima corresponding to thermal hills of approximately 10–15 pixels in diameter in the temperature image *T* (Figure 8, top left). Our strategy relies on the usage of mathematical morphology operators [34] to suppress large-scale structures in the temperature image *T* while preserving localized warm patches corresponding to the thermal markers.

First, a background temperature image, $T_{\mathrm{bg}}$, is obtained through an opening operation $O_{\mathrm{SE}}\left(T\right)$ composed of an erosion $E_{\mathrm{SE}}\left(T\right)$ followed by a dilation $D_{\mathrm{SE}}\left(T\right)$, both using the same structuring element SE [34]. We use a disk-shaped structuring element SE whose radius is chosen about twice (in pixels) the expected radius of the thermal markers. The erosion operator strongly attenuates the small thermal structures caused by the markers and the dilation restores the size of the large-scale structures remaining in $T_{\mathrm{bg}}$ (Figure 8, top right). In practice, these morphological operations are performed with the MATLAB functions strel, imerode and imreconstruct from the “Image Processing” toolbox.

The thermal relief image containing the small-scale features is obtained by subtracting the background from the original temperature image:

(25) $$T_{\mathrm{markers}}\left(x,y\right)=T\left(x,y\right)-T_{\mathrm{bg}}\left(x,y\right).$$

$T_{\mathrm{markers}}$ represents the local temperature excess relative to the surrounding background. In this representation, each heated sphere appears as a positive thermal hill (Figure 8, bottom left).

#### Selection and Localization of the Thermal Markers

Once $T_{\mathrm{markers}}$ are obtained, the second step of the processing sequence is to detect the hot patches corresponding to the thermal markers. A binary image $B_{\mathrm{markers}}$ is constructed by hard thresholding:

(26) $$B_{\mathrm{markers}}\left(x,y\right)=\left\{\begin{array}{cc} 1, & \mathrm{if}\;T_{\mathrm{markers}}\left(x,y\right)\geq \Delta T_{\min},\\ 0, & \mathrm{otherwise}. \end{array}\right.$$

where Δ*T*_min_ = 2 °C.

The binary mask $B_{\mathrm{markers}}$ is denoised by applying a sequence of standard morphological operators [34]:

- *Area opening:* to remove small spurious regions,
  (27) $$B_{\mathrm{markers}}↢\mathrm{AreaOpen}\left(B_{\mathrm{markers}};A_{\min}^{\left(0\right)}\right),$$
  where $A_{\min}$ is a small area threshold (e.g., 20 pixels).
- *Closing:* with a small disk structuring element $S_{c}$ to connect fragmented parts,
  (28) $$B_{\mathrm{markers}}↢\mathrm{Close}\left(B_{\mathrm{markers}};S_{d}\right),$$
  where $S_{d}$ is a disk-shaped structural element with a small radius (e.g., 2 pixels).
- *Hole filling:* to produce compact regions,
  (29) $$B_{\mathrm{markers}}↢\mathrm{FillHoles}\left(B_{\mathrm{markers}}\right).$$

These operations are performed with the MATLAB functions bwareaopen, imclose and imfill from the “Image Processing” toolbox.

Further processing of $B_{\mathrm{markers}}$ given by Equation (29) is needed to obtain a reliable localization of the thermal markers. The characteristic marker diameter is known (10–15 pixels). For each connected component $C_{k}$, we compute:

(30) $$A_{k}=\left|C_{k}\right|,\;P_{k}=\mathrm{Perimeter}\left(C_{k}\right),$$

where |·| denotes the number of pixels. We retain components whose area lies within a plausible range derived from the expected radius $r\in [r_{\min},r_{\max}]$ (with $r_{\min}=d_{\min}/2$ and $r_{\max}=d_{\max}/2$):

(31) $$A_{\min}\leq A_{k}\leq A_{\max},$$

with

(32) $$A_{\min}=\alpha_{\min}\pi r_{\min}^{2},\;A_{\max}=\alpha_{\max}\pi r_{\max}^{2},$$

where $\alpha_{\min}<1$ and $\alpha_{\max}>1$ allow for irregular boundaries (typical values: $\alpha_{\min}=0.4$ and $\alpha_{\max}=2.5$).

Optionally, we compute a circularity descriptor to reject elongated artefacts:

(33) $${\mathrm{circ}}_{k}=\frac{4\pi A_{k}}{P_{k}^{2}+\varepsilon },$$

where ε is a small constant to avoid division by zero. Components are retained if ${\mathrm{circ}}_{k}\geq \tau_{\mathrm{circ}}$ (e.g., $\tau_{\mathrm{circ}}=0.20$), which remains permissive for diffuse hills.

Additionally, we enforce the physical contrast condition at the component level by requiring a sufficiently high peak relief:

(34) $$R_{k}^{\max}=\max_{\left(x,y\right)\in C_{k}}R\left(x,y\right)\geq \Delta T_{\min}.$$

The final binary mask is the union of the retained components:

(35) $$\mathrm{BW}\left(x,y\right)=\underset{k\in K_{\mathrm{keep}}}{⋁}1_{C_{k}}\left(x,y\right).$$

For each retained component $C_{k}$, the marker location is estimated by a weighted barycenter (weighted centroid) using the relief map *R* as weights. Let the pixel coordinates be $\left(x_{i},y_{i}\right)$ for $\left(x_{i},y_{i}\right)\in C_{k}$ and weights $w_{i}=R\left(x_{i},y_{i}\right)$. The weighted barycenter is:

(36) $${\hat{x}}_{k}=\frac{\sum_{\left(x_{i},y_{i}\right)\in C_{k}}x_{i}\;w_{i}}{\sum_{\left(x_{i},y_{i}\right)\in C_{k}}w_{i}},\;{\hat{y}}_{k}=\frac{\sum_{\left(x_{i},y_{i}\right)\in C_{k}}y_{i}\;w_{i}}{\sum_{\left(x_{i},y_{i}\right)\in C_{k}}w_{i}}.$$

This estimator is robust to diffuse boundaries and moderate anisotropy: pixels closer to the hill summit contribute more strongly than peripheral pixels whose apparent temperature is reduced by angular emissivity effects.

We then extract connected components ${\left\{C_{k}\right\}}_{k=1}^{K}$ from ${\mathrm{BW}}_{3}$ using 8-connectivity and applying standard morphological cleaning operations. Connected components are then extracted. Candidate regions are filtered according to their area, and only components whose equivalent diameter falls within the expected size range of 10–15 pixels are retained (Figure 8, bottom right). This constraint efficiently removes spurious detections caused by noise or residual background structures.

For each validated thermal hill, the marker position is defined as the weighted barycenter of the corresponding connected component, using the relief image $T_{\mathrm{markers}}\left(x,y\right)$ as a weighting function. This choice provides a physically meaningful and sub-pixel accurate localization that is robust to diffuse boundaries and moderate asymmetry induced by emissivity effects or by the proximity of the heating wire.

### 6.4. Matching of Homologous Targets Between VIS and LWIR Images (Algorithm A4)

After independent detection of the targets in the visible and LWIR images, reliable one-to-one correspondences must be established between the two point sets. This task is complicated by three factors: (i) some targets may be missing in one modality, (ii) the spatial arrangement of the targets exhibits strong geometric symmetries, and (iii) the two sensors have significantly different spatial resolutions. The proposed method addresses these issues by combining robust estimation with physically motivated constraints derived from the acquisition setup.

Let ${\left\{p_{i}^{\mathrm{IR}}\right\}}_{i=1}^{M}$ and ${\left\{p_{j}^{\mathrm{VIS}}\right\}}_{j=1}^{N}$ denote the sets of detected target centres in the LWIR and visible images, respectively. The objective is to identify a subset of one-to-one correspondences between these sets while allowing for non-matched points, and to estimate a geometric transformation mapping LWIR coordinates onto visible coordinates.

To reduce the search space while remaining robust to missing points and symmetries, local geometric signatures are used. For each target, a signature is defined as the ordered distances to its nearest neighbours within the same modality. These signatures are normalized to reduce sensitivity to scale differences between the two images. For each LWIR target, a shortlist of candidate visible targets with the most similar signatures is retained.

Correspondences and the geometric transformation are estimated jointly using a RANSAC-based approach [35]. At each iteration, triplets of LWIR targets are randomly sampled and paired with candidate visible triplets drawn from the corresponding shortlists. An affine transformation is then estimated from each hypothesis and evaluated against the full set of detected targets.

Because the cameras are rigidly mounted with nearly parallel optical axes, mirrored correspondences are physically impossible. This constraint is enforced in two ways. First, the orientation of each sampled point triplet is preserved: the sign of the oriented area of the triangle formed by the LWIR points must match that of the corresponding visible points. Second, affine transformations whose linear part has a negative determinant are rejected, as they correspond to reflections.

For each valid transformation hypothesis, all LWIR targets are projected into the visible image. One-to-one assignment is then performed using a distance-based cost matrix, with explicit handling of non-assigned points. During the RANSAC phase, relatively permissive distance thresholds are used to accommodate imperfect hypotheses.

The fixed mechanical configuration of the system implies that the image axes of the visible and LWIR cameras are approximately parallel. This physical property is encoded as a monotonicity constraint on the correspondences. Specifically, the relative ordering of matched targets along the horizontal and vertical axes must be preserved between modalities. This condition is quantified using Spearman rank correlation coefficients computed independently for the *x* and *y* coordinates of the matched pairs. Hypotheses that violate this constraint are rejected, as they typically correspond to crossing or geometrically implausible matches.

Once the best hypothesis has been identified, the affine transformation is refined using all inlier correspondences. A final assignment step is then performed with stricter distance and monotonicity thresholds to obtain the definitive set of homologous point pairs. This two-stage strategy ensures robustness to missing detections while providing accurate alignment.

The transformation is optimized to provide maximal accuracy within the region delimited by the thermal markers. Outside this region, extrapolation errors may occur due to perspective effects and unmodeled distortions. This limitation is acceptable in the present application, as the anatomical regions of interest are always located within the marker-defined domain.

By combining local geometric descriptors, robust estimation, and physically motivated constraints reflecting the fixed and nearly parallel camera configuration, the proposed method achieves stable and accurate matching of homologous targets between visible and LWIR images. This approach effectively suppresses mirrored and crossing correspondences and provides reliable geometric alignment within the clinically relevant region.

### 6.5. Registration of VIS and LWIR Images

**Figure 9:**
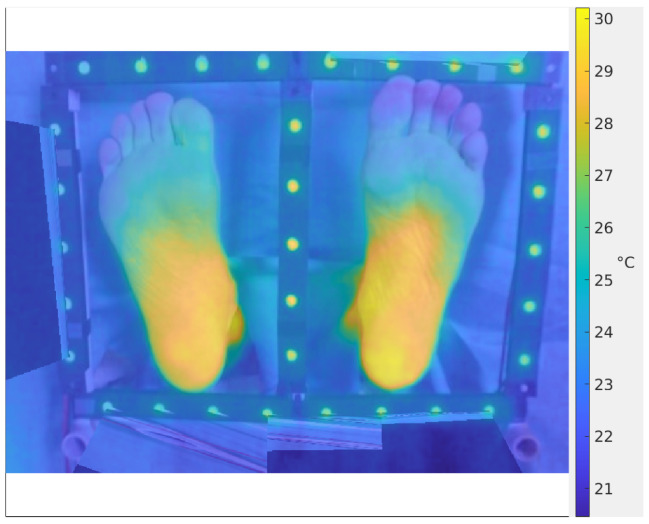
Final image merging the VIS and LWIR data obtained at the end of the processing chain described in the present study.

The pairs of marker centres detected as explained above are used to build an anamorphosis model to superimpose the VIS image onto the LWIR image. This can easily be performed using the MATLAB functions fitgeotform2d and imwarp. Both the VIS and LWIR temperature images may be merged to obtain the final image to be analysed by the physicians (Figure 9). As can be observed, the fact that the patient’s feet are surrounded by thermal markers makes the registration accurate. Conversely, the registration is poor outside of the inner domain delimited by the marker because the anamorphosis model is poorly constrained.

## 7. Discussion

The present study shows that quantitative and geometrically registered plantar thermal and visible images can be obtained with a low-cost LWIR camera like the FLIR C5, provided that the acquisition geometry and the radiometric processing are controlled. The proposed workflow makes explicit the full measurement chain, from bedside acquisition constraints to the generation of temperature images suitable for interpretation. A consumer-grade LWIR camera can reach high measurement repeatability when (i) environmental parameters are recorded, (ii) camera settings are standardized, and (iii) post-processing corrects the systematic errors caused by vignetting.

A key issue is the choice to perform vignetting correction in the radiometric domain, prior to temperature inversion. Because detector and optical non-uniformities are intrinsically multiplicative in radiance space, correcting them before applying the non-linear Planck inversion avoids temperature-dependent residuals and reduces bias over scenes spanning several degrees. The gain-field model derived from repeated flat-field acquisitions provides a practical compromise between physical consistency and hospital feasibility, and it can be updated periodically to account for camera aging.

Among the processing steps detailed in the present study, the vignetting correction is the only one that produces an amplification of the noise level in the final thermography as shown in Figure 9. This comes from Equation (12) where a noisy radiometric image is divided by a noisy master radiometric gain. The resulting noise translates into a global accuracy of 0.15 °C in the temperature images. This value was experimentally derived from statistics we made with our data set. Let us remark that these good statistics are obtained provided we have a sufficient number (e.g., $N_{k}\geq 16$ in Equation (3)) of flat-field images in Equation (4). The examples of flat-fields displayed in Figure 4 show that the vignetting artefact may strongly vary from one device to another. However, we checked that, for a given camera, the vignetting master gain is not affected by ambient temperature changes.

The second major practical challenge addressed in the present study is the VIS–LWIR registration at short working distances. Parallax effects and distinct lens distortions prevent the default in-camera overlay from being sufficiently accurate for clinical use. The proposed calibration using thermal markers and matching strategy yields a stable mapping between VIS and LWIR images in the region of interest, enabling physicians to localize anatomical landmarks on LWIR images. Since the registration is based solely on the thermal markers, there is no influence of possible deformities or amputated parts of the patient’s feet. This step is essential for routine use because interpretability often depends on the ability to associate thermal anomalies with specific plantar zones recognized in the VIS images.

We emphasize that the registration procedure proposed in the present paper is designed to have the smallest-as-possible effect on the LWIR images and that the main geometric transform determined with the thermal markers (Section 6.1) is applied on the VIS images. The accuracy of the registration strongly depends on the accuracy of the localization of the centres of the thermal markers, particularly in the LWIR images where the markers appear as diffuse thermal hills (Figure 8). Figure 9 shows that the VIS–LWIR registration is rather good in the inner domain limited by the thermal markers and poor outside where the anamorphosis model is badly constrained. We experimentally found that the registration error in the inner domain is less than 2 mm. This rather small value is reachable provided the thermal markers are located near the plane that passes through the soles of the patient’s feet. If the markers are too far from this plane (i.e., typically more than 4 cm), the parallax effects will not be correctly estimated, leading to a registration error that may exceed 5 mm.

The algorithms detailed in the present paper rely on the availability of the radiometric data embedded in the jpeg images produced by the FLIR C5 camera. These algorithms may be used with data obtained with cameras other than the FLIR C5, provided these cameras are radiometric. The calibrations described in Section 4.1 for flat-field and Section 5 for the geometric distortion must be performed for each camera. Periodic flat-field acquisitions are recommended to account for eventual drift of the camera.

A conversion of the processed data to a DICOM-compatible format is envisaged to facilitate integration into hospital information systems and clinical workflows. This will constitute an important step towards the integration of diabetic foot thermography into a multimodal approach involving data from different medical specialities (Doppler ultrasound, TcPO2, RMN, etc.).

## 8. Conclusions

The present study shows that low-cost medical thermography may be useful for diabetic foot evaluation, provided that the low financial cost of the equipment is supplemented by a significant investment in data processing (advanced radiometric processing, vignetting correction, registration) and rigorous protocols are implemented (rigid frame, thermal markers, reference blackbody, fixed distance). We believe that future quality assurance guidelines (such as those of the American Academy of Thermology [36] or the International Academy of Clinical Thermology [37,38]) will evolve to certify not only the hardware, but also the combination of “hardware + software” where a rigorous clinical protocol would depend on the accuracy of software corrections. These conditions are essential to ensure the reproducibility, clinical relevance, and interpretability of thermal measurements by clinicians.

The various processing steps presented in this article constitute the basic processing package for producing VIS and LWIR images corrected for various artefacts and that can be overlaid (Figure 9). These images can then be processed in different ways (segmentation, contrast enhancement, etc.) during the medical interpretation phase. These processing steps will be the subject of a forthcoming article.

## Data Availability

The original contributions presented in this study are included in the article. Further inquiries can be directed to the corresponding author.

## Appendix A. Radiometric Vignetting Model

**Algorithm A1** Build a Radiometric Vignetting (Gain-Field) Model from Multiple Flat-Field Epochs
Require: Epoch list $E={\left\{E_{k}\right\}}_{k=1}^{K}$, each with name, optional time, and flats (matrices or filenames) Require: Options: Gaussian scale σ (auto if empty), time aggregation median/mean, clamp quantiles $\left(q_{lo},q_{hi}\right)$, normalization mode (mean/median/center), loader function L(·), StabilityReport flag Ensure: Model with per-epoch gains $\left\{G_{k}\right\}$, master gain $G^{master}$, names/times, stability metrics 1: Initialize empty list of gain fields ${\left\{G_{k}\right\}}_{k=1}^{K}$ and names $\left\{n_{k}\right\}$ 2: **for** k←1 to *K* **do** 3: $n_{k}\leftarrow E_{k}.\mathrm{name}$; store $t_{k}\leftarrow E_{k}.\mathrm{time}$ if available 4: **Load stack:** read each flat in $E_{k}.\mathrm{flats}$ using L if it is a filename 5: Form 3D stack $I^{\left(k\right)}\in R^{H\times W\times N_{k}}$ 6: **Time aggregation:** $I\leftarrow \mathrm{Agg}(I^{\left(k\right)})$ where Agg=median(·,3) or mean(·,3) 7: **Robust clamping:** $l\leftarrow Q(I,q_{lo})$, $h\leftarrow Q(I,q_{hi})$, I←min(max(I,l),h) 8: **if** σ is empty **then** 9: $\sigma_{k}\leftarrow \max\;\left(10,\;\mathrm{round}\;\left(\min(H,W)/10\right)\right)$ 10: **else** 11: $\sigma_{k}\leftarrow \sigma$ 12: **end if** 13: **Slow-field extraction:** $I_{LP}\leftarrow \mathrm{GaussianFilter}\left(I,\sigma_{k}\right)$ 14: **Gain normalization:** $$r\leftarrow \left\{\begin{array}{cc} \mathrm{mean}(I_{LP}) & \text{if norm = mean}\\ \mathrm{median}(I_{LP}) & \text{if norm = median/center} \end{array}\right.\;G_{k}\leftarrow \max\left(\frac{I_{LP}}{r},10^{-6}\right)$$ 15: **end for** 16: **Master gain (robust across epochs)** 17: Stack gains $G_{\mathrm{stack}}\left(:,:,k\right)\leftarrow G_{k}$ and compute $$G^{master}\leftarrow \max\;\left(\mathrm{median}\left(G_{\mathrm{stack}},3\right),\;10^{-6}\right)$$ 18: **Inter-epoch stability metrics (multiplicative)** 19: **for** i←1 to *K* **do** 20: **for** j←1 to *K* **do** 21: $\Delta_{ij}\leftarrow \log\left(G_{i}\right)-\log\left(G_{j}\right)$ 22: ${\mathrm{RMS}}_{ij}\leftarrow \sqrt{\mathrm{mean}(\Delta_{ij}^{2})}$ 23: ${\mathrm{MAX}}_{ij}\leftarrow \max(|\Delta_{ij}\left|\right)$ 24: $c_{ij}\leftarrow \mathrm{corr}\;\left(\log\left(G_{i}\right)-\bar{\log(G_{i})},\;\log\left(G_{j}\right)-\bar{\log(G_{j})}\right)$ 25: **end for** 26: **end for** 27: **Assemble model** 28: model.gainFields $\leftarrow \{G_{k}\}$; model.masterGain $\leftarrow G^{master}$; model.names $\leftarrow \{n_{k}\}$; model.times$\leftarrow \{t_{k}\}$ 29: model.metrics.rmsLog $\leftarrow [{\mathrm{RMS}}_{ij}]$; model.metrics.maxAbsLog $\leftarrow [{\mathrm{MAX}}_{ij}]$; model.metrics.corr$\leftarrow [c_{ij}]$ 30: **if** StabilityReport is true **then** 31: Display stability figures (RMS map, correlation map, master gain) 32: **end if** 33: **return** model

## Appendix B. Radiometric Vignetting Correction

**Algorithm A2** Radiometric Vignetting (Gain-Field) Correction with Optional Time-Dependent Gain Selection
Require: Radiometric image $S\in R^{H\times W}$; model containing a master gain field $G^{\mathrm{master}}$ and optionally time-stamped gain fields ${\left\{\left(t_{k},G_{k}\right)\right\}}_{k=1}^{K}$ Require: Options: gain mode GainMode∈{master,nearest,interp}; acquisition time *t* (optional); gain floor ε>0 Ensure: Corrected image $S_{\mathrm{corr}}$ and diagnostics info 1: Convert S←double(S) 2: mode←lower(GainMode) 3: **Gain-field selection** 4: **if** mode=master **then** 5: $G\leftarrow G^{\mathrm{master}}$; used← master 6: **else** 7: **if** time series $\left\{t_{k}\right\}$ is missing **or** *t* is not provided **then** 8: $G\leftarrow G^{\mathrm{master}}$; used← fallback 9: **else** 10: Sort gain fields by time: $\left(t_{k},G_{k}\right)\leftarrow \mathrm{sort}\left(\left\{\left(t_{k},G_{k}\right)\right\}\right)$ 11: **if** mode=nearest **then** 12: $k^{★}\leftarrow \arg\min_{k}\left|t_{k}-t\right|$ 13: $G\leftarrow G_{k^{★}}$; used← nearest 14: **else if** mode=interp **then** 15: **if** $t\leq t_{1}$ **then** 16: $G\leftarrow G_{1}$ 17: **else if** $t\geq t_{K}$ **then** 18: $G\leftarrow G_{K}$ 19: **else** 20: Find *j* such that $t_{j}\leq t<t_{j+1}$ 21: $w\leftarrow \frac{t-t_{j}}{t_{j+1}-t_{j}}$ 22: ▹ Log-domain interpolation is appropriate for multiplicative gain fields 23: $G\leftarrow \exp\;\left(\left(1-w\right)\log\left(G_{j}\right)+w\log\left(G_{j+1}\right)\right)$ 24: **end if** 25: used ← interp 26: **else** 27: **error** (invalid gain mode) 28: **end if** 29: **end if** 30: **end if** 31: **Safe division and correction** 32: G←max(G,ε) ▹ element-wise floor to avoid division by zero 33: $S_{\mathrm{corr}}\leftarrow S⊘G$ ▹ element-wise division 34: **Diagnostics** 35: Fill info with: selected mode used, statistics of *G*, *S*, and $S_{\mathrm{corr}}$ (min/max/mean/median) 36: **return**$S_{\mathrm{corr}}$, info

## Appendix C. Thermal Marker Detection

**Algorithm A3** Detection of Warm “Hills” on a Heterogeneous Cold Background (Morphological Approach)
Require: Temperature image $T\in R^{H\times W}$, typical hill diameter $d_{\mathrm{patch}}$ (pixels), minimum contrast $\Delta T_{\min}$ (e.g., 2 °C) Ensure: Weighted centroids C, region properties P, binary maxima mask *M*, relief image *R*, background estimate *B* 1: Convert T←double(T) 2: $r\leftarrow d_{\mathrm{patch}}/2$ ▹ expected hill radius 3: **Background estimation by morphological reconstruction** 4: Compute marker image by erosion: $$T_{\mathrm{marker}}\leftarrow \varepsilon_{S}\left(T\right),\;S=\mathrm{disk}\left(\mathrm{round}(2r)\right)$$ 5: Reconstruct background (opening by reconstruction): $$B\leftarrow \mathrm{Rec}\left(T_{\mathrm{marker}},\;T\right)$$ 6: Compute relief above background: R←T−B 7: **Candidate maxima extraction** 8: Suppress maxima shallower than $\Delta T_{\min}$ and detect regional maxima: $$M\leftarrow \mathrm{RegionalMax}\;\left(\mathrm{HMax}(R,\Delta T_{\min})\right)$$ 9: Dilate candidate mask to cover hill extent: $$M\leftarrow \delta_{\mathrm{disk}(\mathrm{round}(r))}\left(M\right)$$ 10: Compute a global relief scale $R_{\max}\leftarrow \max\left(R\right)$ and a conservative threshold $$\tau \leftarrow \max\;\left(\Delta T_{\min},\;0.1\times 0.5\;R_{\max}\right)$$ 11: Enforce relief threshold: M←M∧(R>τ) 12: **Mask cleaning** 13: Remove small components: $$M\leftarrow \mathrm{AreaOpen}\;\left(M,\;\mathrm{round}\;\left(0.2\pi r^{2}\right)\right)$$ 14: Morphological closing with small disk (radius 2 px): M←Close(M,disk(2)) 15: Fill holes: M←FillHoles(M) 16: **Region extraction and weighted centroids** 17: Compute connected components: ${\left\{\Omega_{k}\right\}}_{k=1}^{K}\leftarrow \mathrm{ConnComp}\left(M\right)$ (8-connectivity) 18: For each component $\Omega_{k}$, compute properties on the relief image *R*: area $A_{k}$, centroid $c_{k}$, weighted centroid $w_{k}$, maximum intensity $I_{k}^{\max}$ 19: Collect region properties $P\leftarrow {\left\{\;\left(A_{k},c_{k},w_{k},I_{k}^{\max}\right)\;\right\}}_{k=1}^{K}$ 20: **if** K=0 **then** 21: C←∅ 22: **else** 23: $C\leftarrow {\left\{w_{k}\right\}}_{k=1}^{K}$ ▹ output centroids = weighted centroids 24: **end if** 25: **return** C,P,M,R,B

## Appendix D. LWIR–Visible Target Matching

**Algorithm A4** Robust LWIR–Visible Target Matching
Require: LWIR points $P^{\mathrm{IR}}$, Visible points $P^{\mathrm{VIS}}$, parameters $\left(K,L,N_{\mathrm{it}},\tau_{d}^{s},\tau_{d}^{h},\rho^{s},\rho^{h},\lambda \right)$ Ensure: Matched pairs C, affine transform $T_{\mathrm{IR}\to \mathrm{VIS}}$ 1: **Candidate selection** 2: Compute local neighbor-distance signatures in each modality 3: For each LWIR point, retain *L* visible candidates with closest signatures 4: **RANSAC with soft constraints** 5: $S^{★}\leftarrow -\infty$, $T^{★}\leftarrow \emptyset$, $C^{★}\leftarrow \emptyset$ 6: **for** t←1 **to** $N_{\mathrm{it}}$ **do** 7: Sample 3 LWIR points and 3 distinct visible candidates 8: **if** triangle orientations differ **then** 9: **continue** ▹ anti-mirror 10: **end if** 11: Estimate affine transform *T* 12: **if** $\det(A_{T})<0$ **then** 13: **continue** ▹ anti-reflection 14: **end if** 15: Project all LWIR points using *T* 16: Solve one-to-one assignment with null matches (penalty λ) 17: Keep inliers with distance $<\tau_{d}^{s}$ 18: **if** fewer than 3 inliers **then** 19: **continue** 20: **end if** 21: Compute Spearman rank correlations $\rho_{x}$ and $\rho_{y}$ on inlier pairs 22: **if** $\min\left(\rho_{x},\rho_{y}\right)<\rho^{s}$ **then** 23: **continue** 24: **end if** 25: Compute score from inlier count and residual error 26: **if** score improves **then** 27: Update $S^{★}$, $T^{★}$, and $C^{★}$ 28: **end if** 29: **end for** 30: **if** $T^{★}=\emptyset$ **then** 31: **return** failure 32: **end if** 33: **Refinement** 34: Re-estimate affine transform $T_{\mathrm{IR}\to \mathrm{VIS}}$ using $C^{★}$ 35: **if**$\det(A_{T_{\mathrm{IR}\to \mathrm{VIS}}})<0$ **then** 36: **return** failure 37: **end if** 38: **Final matching with hard constraints** 39: Project LWIR points using $T_{\mathrm{IR}\to \mathrm{VIS}}$ 40: Solve assignment and retain pairs with distance $<\tau_{d}^{h}$ 41: Compute $\rho_{x}$ and $\rho_{y}$ on final pairs 42: **if**$\min\left(\rho_{x},\rho_{y}\right)<\rho^{h}$ **then** 43: **return** failure 44: **end if** 45: **return** C and $T_{\mathrm{IR}\to \mathrm{VIS}}$

## References

[1] Zimmet P.Z., Magliano D.J., Herman W.H., Shaw J.E. (2014). Diabetes: A 21st century challenge. Lancet Diabetes Endocrinol..

[2] Forbes J.M., Cooper M.E. (2013). Mechanisms of diabetic complications. Physiol. Rev..

[3] Schulze M.B., Hu F.B. (2005). Primary prevention of diabetes: What can be done and how much can be prevented?. Annu. Rev. Public Health.

[4] Faus Camarena M., Izquierdo-Renau M., Julian-Rochina I., Arrébola M., Miralles M. (2023). Update on the use of infrared thermography in the early detection of diabetic foot complications: A bibliographic review. Sensors.

[5] Sivakumar D.T., Murray B., Moore Z., Patton D., O’Connor T., Avsar P. (2024). Can thermography predict diabetic foot ulcer risk in patients with diabetes mellitus? A systematic review. J. Tissue Viability.

[6] Castillo-Morquecho R., Guevara E., Ramirez-GarciaLuna J.L., Martínez-Jiménez M.A., Medina-Rangel M.G., Kolosovas-Machuca E.S. (2024). Digital infrared thermography and machine learning for diabetic foot assessment: Thermal patterns and classification. J. Diabetes Metab. Disord..

[7] Zakaria S.A., Low C.L., Kow R.Y., Mohamad Z.Z., Abidin M.R., Ahmad A.C., Ahmad M.W., Zulkifly A.H., Sulaiman A.S. (2024). Thermography research in diabetic foot: Insights from a Scopus-based bibliometric study. Cureus.

[8] Haris R., Marappan R. (2025). Bio-Inspired DFU-ThermoNet: A Spiking Transformer-Driven Framework for Early Diagnosis and Risk Prediction of Diabetic Foot Ulcers Using Thermal Imaging. IEEE Access.

[9] Cao Z., Zeng Z., Xie J., Zhai H., Yin Y., Ma Y., Tian Y. (2023). Diabetic plantar foot segmentation in active thermography using a two-stage adaptive gamma transform and a deep neural network. Sensors.

[10] Wu L., Huang R., He X., Tang L., Ma X. (2024). Advances in Machine Learning-Aided Thermal Imaging for Early Detection of Diabetic Foot Ulcers: A Review. Biosensors.

[11] Alwashmi M.F., Alghali M., AlMogbel A., Alwabel A.A., Alhomod A.S., Almaghlouth I., Temsah M., Jamal A. (2025). The Use of AI-Powered Thermography to Detect Early Plantar Thermal Abnormalities in Patients with Diabetes: Cross-Sectional Observational Study. JMIR Diabetes.

[12] Ilo A., Romsi P., Mäkelä J. (2020). Infrared thermography and vascular disorders in diabetic feet. J. Diabetes Sci. Technol..

[13] Nguyen T.X.B., Rosser K., Chahl J. (2021). A review of modern thermal imaging sensor technology and applications for autonomous aerial navigation. J. Imaging.

[14] Kesztyüs D., Brucher S., Wilson C., Kesztyüs T. (2023). Use of infrared thermography in medical diagnosis, screening, and disease monitoring: A scoping review. Medicina.

[15] Joshi V., Manivannan N., Jarry Z., Carmichael J., Vahtel M., Zamora G., Calder C., Simon J., Burge M., Soliz P. Low cost thermal camera for use in preclinical detection of diabetic peripheral neuropathy in primary care setting. Proceedings of the Optics and Biophotonics in Low-Resource Settings IV.

[16] Marcon Alfieri F., Aquino dos Santos A.C., da Silva Dias C., Rizzo Battistella L. (2023). The concordance study of the portable camera FLIR C5 for detecting asymmetry of skin temperature in patients with stroke sequelae. Thermol. Int..

[17] Raj A., Sadhana K., Suhaas K.P. (2024). A novel multi-modal approach that fuses dermoscopic images with thermal imaging in pre-emptive identification of diabetic foot ulcers (DFUs). SN Comput. Sci..

[18] Wartakusumah R., Yamada A., Noguchi H., Oe M. (2025). Analysis of foot thermography images of diabetic patients using artificial intelligence: A scoping review. Diabetes Res. Clin. Pract..

[19] Goyal M., Reeves N.D., Rajbhandari S., Ahmad N., Wang C., Yap M.H. (2020). Recognition of ischaemia and infection in diabetic foot ulcers: Dataset and techniques. Comput. Biol. Med..

[20] Fahmy A.M. (2025). Assessing the Efficacy of AI Models in Medical Imaging Analysis for the Early Identification of Diabetic Foot Ulcers and Gangrene: A Review. Prem. J. Sci..

[21] Alfieri F.M., da Silva Dias C., dos Santos A.C.A., Battistella L.R. (2020). Comparison of sensitivity and plantar cutaneous temperature of patients with stroke and Diabetes Mellitus: A pilot case-control study. Technol. Health Care.

[22] https://www.flir.com/products/c5/.

[23] https://www.flir.com/discover/professional-tools/what-is-msx/.

[24] Steketee J. (1973). Spectral emissivity of skin and pericardium. Phys. Med. Biol..

[25] Dell’Isola G.B., Cosentini E., Canale L., Ficco G., Dell’Isola M. (2021). Noncontact Body Temperature Measurement: Uncertainty Evaluation and Screening Decision Rule to Prevent the Spread of COVID-19. Sensors.

[26] Marins J.C.B., Moreira D.G., Cano S.P., Quintana M.S., Soares D.D., de Andrade Fernandes A., da Silva F.S., Costa C.M.A., dos Santos Amorim P.R. (2014). Time required to stabilize thermographic images at rest. Infrared Phys. Technol..

[27] Larobina M. (2023). Thirty years of the DICOM standard. Tomography.

[28] Vardasca R., Tereso M., Pratas A., Alturas B., Martinho D., Bento F., Guarda T., Portela F., Diaz-Nafria J.M. (2024). Integration proposal for thermal imaging modality into health information systems. Advanced Research in Technologies, Information, Innovation and Sustainability: Third International Conference, ARTIIS 2023, Proceedings; Communications in Computer and Information Science.

[29] Yuan W., Hua W. (2022). A case study of vignetting nonuniformity in UAV-Based uncooled thermal cameras. Drones.

[30] Tendero Y., Gilles J. (2024). ADMIRE: A locally adaptive single-image, non-uniformity correction and denoising algorithm: Application to uncooled IR camera. arXiv.

[31] Minkina W., Dudzik S. (2009). Infrared Thermography: Errors and Uncertainties.

[32] Fetić A., Jurić D., Osmanković D. The procedure of a camera calibration using Camera Calibration Toolbox for MATLAB. Proceedings of the IEEE 2012 35th International Convention MIPRO.

[33] Vidas S., Lakemond R., Denman S., Fookes C., Sridharan S., Wark T. (2012). A mask-based approach for the geometric calibration of thermal-infrared cameras. IEEE Trans. Instrum. Meas..

[34] Hÿtch M., Peter W., Hawkes P.W. (2020). Morphological Image Operators.

[35] Fischler M.A., Bolles R.C. (1981). Random sample consensus: A paradigm for model fitting with applications to image analysis and automated cartography. Commun. ACM.

[36] https://www.aathermology.org/.

[37] https://www.iact-org.org/.

[38] Ring E.F.J., Ammer K. (2015). The technique of infrared imaging in medicine. Infrared Imaging: A Casebook in Clinical Medicine.

